# Racial differences in treatment and survival among older patients with multiple myeloma

**DOI:** 10.1002/cam4.6915

**Published:** 2024-01-17

**Authors:** Rong Wang, Natalia Neparidze, Xiaomei Ma, Graham A. Colditz, Su‐Hsin Chang, Shi‐Yi Wang

**Affiliations:** ^1^ Department of Chronic Disease Epidemiology Yale School of Public Health New Haven Connecticut USA; ^2^ Cancer Outcomes, Public Policy, and Effectiveness Research (COPPER) Center Yale University New Haven Connecticut USA; ^3^ Department of Internal Medicine Section of Hematology, Yale School of Medicine New Haven Connecticut USA; ^4^ Department of Surgery, Division of Public Health Sciences Washington University School of Medicine St. Louis Missouri USA

**Keywords:** multiple myeloma, racial disparity, SEER‐Medicare, survival, treatment

## Abstract

**Background:**

Treatments for multiple myeloma (MM) have evolved over time and improved MM survival. While racial differences in MM treatment and prognosis between non‐Hispanic African American (NHAA) and non‐Hispanic White (NHW) patients are well‐established, it is unclear whether they have persisted after the introduction of novel agents.

**Methods:**

Using the Surveillance, Epidemiology, and End Results‐Medicare linked database, our study investigated racial difference in the receipt of treatment within 1 year following diagnosis and assessed survival outcomes among Medicare beneficiaries (≥66 years) diagnosed with MM from 2007 to 2017. We applied multivariable Cox proportional hazards models to estimate the association between race and survival and presented hazard ratios (HRs).

**Results:**

Of 2094 NHAA and 11,983 NHW older patients with MM, 59.5% and 64.8% received treatment during the first year, respectively. Discrepancy in the proportion of patients receiving treatment between the two groups increased from 2.9% in 2007 to 2009 to 6.9% in 2014–2017. After controlling for relevant factors, patients who received treatment within the first year had lower mortality than those who did not (HR = 0.90, 95% confidence interval [CI]: 0.86–0.94). NHAA patients had a lower probability to receive treatments during the first year than NHW patients (HR = 0.91, 95% CI: 0.85–0.97) but had lower mortality (HR = 0.94, 95% CI: 0.88–1.00). The lower mortality was only observed among patients who received no treatment (HR = 0.84, 95% CI: 0.77–0.93); NHAA and NHW patients who received treatment had similar survival (*p* = 0.63).

**Conclusions:**

The increasing racial disparity in treatment utilization over time is concerning. Efforts are needed to eliminate the barriers of receiving treatment.

## 1 INTRODUCTION

Multiple myeloma (MM) is the second most common hematologic malignancy with an estimate of 35,730 new cases and 12,590 new deaths in the United States (US) in 2023.^1^ With a median age at diagnosis of 69 years, MM mainly afflicts older adults.^2^ It is known that there are racial disparities in MM treatment and prognosis with Non‐Hispanic African American (NHAA) individuals have a twofold increased risk to develop and die from MM than their non‐Hispanic White (NHW) counterparts.^2^, ^3^ Furthermore, among NHAA individuals, MM is not only one of the most commonly diagnosed cancers but also a major contributor of cancer death.^4^

Advances in medication development have transformed the therapeutic landscape of MM over the past decades.^5^ Treatment options for MM have expanded beyond chemotherapy and autologous stem‐cell transplant to a list of new therapeutic agents, including immunomodulatory drugs, proteasome inhibitors, monoclonal antibodies, small molecule targeted agents, and lately, antibody drug conjugate and chimeric antigen receptor T‐cell therapy.^6^ The utilization of these novel drugs has improved MM patients' survival. The 5‐year relative survival of MM in the United States has increased from 35% in 2000 to 58% in 2019.^2^ However, previous studies have reported that treatment patterns of MM vary by race.^7^, ^8^, ^9^ Fewer NHAA patients received systemic treatment^7^ and had lower utilization of lenalidomide than their NHW counterparts.^8^ While these studies presented important data that have clear clinical implications, they did not fully examine the treatment patterns and outcomes for patients with MM in the era of novel agents.

To provide a timely evaluation of the treatment patterns and survival of MM among older patients, we assessed racial differences in the receipt of treatment, timing of treatment initiation, and survival between NHAA and NHW Medicare beneficiaries with MM in the real‐world settings. We hypothesized that compared to NHM patients, fewer NHAA patients received treatment for MM in the first year after diagnosis and would also initiate treatment later. As the novel agents have evolved over time, we further hypothesized that more recently diagnosed older patients had better survival, and this temporal difference occurred only among patients who received treatment but not among those who did not.

## 2 METHODS

This study used the Surveillance, Epidemiology, and End Results (SEER)‐Medicare database. The SEER‐Medicare links patient‐level cancer information of Medicare beneficiaries from SEER registries with individual‐level Medicare claims, including claims for inpatient, outpatient, physician services, hospice care, home health agencies, durable medical equipment, and prescription drugs.^10^ The Yale Human Investigation Committee determined that this study did not directly involve human subjects.

This study identified NHAA and NHW patients with newly diagnosed MM via International Classification of Diseases for Oncology, third edition code of 9732. Patients included were ≥66 years at diagnosis during 2007–2017, had no previous cancer diagnosis, enrolled Medicare Parts A & B continuously, and never enrolled health maintenance organizations from 12 months before diagnosis to death or 12/31/2019 that occurred first. Additionally, we excluded patients who were identified by death certificates or autopsy only. Symptomatic MM was defined by a published algorithm.^11^ In brief, we identified hypercalcemia, renal failure, chronic kidney disease, anemia, pathologic fracture, and fracture of vertebral column from inpatient, outpatient, and carrier claims within a 6‐month window around MM diagnosis. As these conditions might be caused by other diseases, to ensure that they were caused by MM, we did not define patients as having CRAB if they had following conditions: (1) hypercalcemia and hyperparathyroidism co‐existed, (2) vertebral fractures and osteoporosis co‐existed, or (3) the patient had anemia or chronic kidney disease more than 6 months before MM diagnosis.^11^

Treatment for MM following within the first year after MM diagnosis was identified through Medicare claims using relevant International Classification of Diseases (ICD) and Healthcare Common Procedure Coding System (HCPCS) codes for injectable drugs and generic names for prescription drugs. MM treatment was defined as receipt of any of the followings: belantamab, bendamustine, bortezomib, carfilzomib, carmustine, cisplatin, cyclophosphamide, daratumumab, doxorubicin, elotuzumab, etoposide, isatuximab, ixazomib citrate, lenalidomide, melphalan, panobinostat, pomalidomide, selinexor, thalidomide, vincristine, unspecified antineoplastic chemotherapy or immunotherapy, or autologous or allogeneic stem‐cell transplant.

Our main endpoint was 5‐year overall survival. To lessen the potential risk of immortal time bias,^12^ we randomly assigned a pseudo‐treatment initiation date for patients who received no MM‐related treatment within the first year after diagnosis. We followed patients from treatment initiation for those who received treatment within the first year after diagnosis, or from pseudo‐treatment initiation for those who did not undergo treatment within the first year, until death, 5 years following treatment initiation, or December 31, 2019, whichever came first. Our secondary outcome was time to initiate treatment. We excluded 712 patients who received no treatment during the first year after diagnosis and died before the pseudo‐treatment initiation date from relevant survival analysis.

We collected information on patient's age at diagnosis, sex, marital status, residential region, state buy‐in (as a proxy marker for socioeconomic status), and percentage of population below poverty at the census tract level. To construct Elixhauser comorbidity score (with adjustment)^13^ and frailty index,^14^, ^15^ we searched for diagnosis codes within 12 months before MM diagnosis and required the codes to be on any inpatient claims or two outpatient/physician claims with an interval exceeding 30 days.^16^ We further adjusted the Elixhauser comorbidity score by excluding conditions that were considered symptoms of MM. The frailty index by Kim et al. uses diagnosis and HCPCS codes over a 12‐month look‐back period and employs a cumulative deficit approach to estimate frailty with beneficiaries being categorized as non‐frail and frail.^14^, ^15^ This approach has been validated in Medicare beneficiaries ≥65 years of age.^14^

We summarized patient characteristics at diagnosis (baseline). For categorical variables, we provided frequencies and percentages and used Pearson's chi‐square test to compare the two racial groups. For continuous variables, we presented their medians and interquartile ranges (IQR). Cochran‐Armitage trend test was used to test trend of treatment by year of diagnosis.

For analyses related to time to initiate treatment in the first year, death within the first year after diagnosis was treated as a competing event. We applied a competing risk model to estimate the cumulative incidence function of treatment initiation and performed the Gray's test to compare cumulative incidence between groups. We implemented a multivariable competing risk regression model using the Fine and Gray method to estimate the adjusted hazard ratios (HRs) and 95% confidence intervals (CIs) for treatment initiation. We included race, the year of MM diagnosis (2007–2009, 2010–2013, and 2014–2017), sex, age at diagnosis (66–69, 70–74, 75–79, 80–84, ≥85 years), marital status (married, unmarried, unknown), region (Northeast, Midwest, South, West), Elixhauser score (0, 1–2, ≥3), frailty index (no, yes), Part D coverage, state buy‐in, and percentage of population below poverty in the census tract in the model.

For analyses focusing on survival, we generated Kaplan–Meier curves and assessed the difference between groups via the log‐rank tests. We utilized multivariable Cox proportional hazards models to assess the association between various factors and survival. The covariates included treatment status along with the previously mentioned covariates. We further stratified patients based on their treatment status (i.e., treated or untreated within 1 year after diagnosis) and followed them from MM diagnosis. Additionally, we carried out a sensitivity analysis on patients with continuous Part D enrollment. Giving the resemblance of the outcomes to those from our primary analyses, we presented findings solely from the main analyses. All statistical tests were two‐sided with a type I error of 0.05 and conducted using SAS Version 9.4 (SAS Institute Inc., Cary, NC).

## 3 RESULTS

**FIGURE 1:**
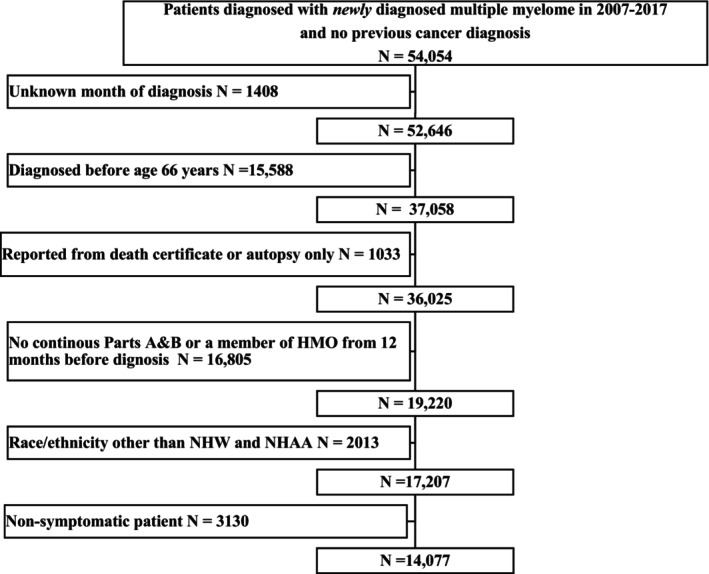
Patients selection flow chart.

The analytic cohort included 14,077 patients (14.9% NHAA and 85.1% NHW) newly diagnosed with symptomatic MM from 2007 to 2017 (Figure 1). We followed NHAA and NHW patients for a median of 1.88 (IQR: 0.44–3.92) years and 2.17 (IQR: 0.50–4.27) years, respectively. The median age of diagnosis was 75 (IQR: 70–81) years for NHAA patients and 77 (IQR: 71–83) years for NHW patients. Compared with NHW patients, NHAA patients were more prone to be female, younger, and unmarried, to have higher Elixhauser and frailty scores, state buy‐in, and Part D enrollment, and to reside in the South and census tracts with a high proportion of population below poverty (all *p*‐values <0.01, Table 1).

**TABLE 1:** Characteristics of newly diagnosed multiple myeloma patients by race and treatment, 2007–2017.

	Overall			Treated			No treatment		
	NHW	NHAA		NHW	NHAA		NHW	NHAA	
	*n* (%)	*n* (%)	*p*	*n* (%)	*n* (%)	*p*	*n* (%)	*n* (%)	*p*
Total	11,983	2094		7760	1245		4223	849	
Treatment									
Never	4223 (35.2)	849 (40.5)	<0.01						
Ever	7760 (64.8)	1245 (59.5)							
Year of diagnosis									
2007–2009	3208 (26.8)	540 (25.8)	0.62	1743 (22.5)	278 (22.3)	0.93	1465 (34.7)	262 (30.9)	0.09
2010–2013	4402 (36.7)	785 (37.5)		2893 (37.3)	471 (37.8)		1509 (35.7)	314 (37.0)	
2014–2017	4373 (36.5)	769 (36.7)		3124 (40.3)	496 (39.8)		1249 (29.6)	273 (32.2)	
Sex									
Female	5505 (45.9)	1233 (58.9)	<0.01	3550 (45.7)	757 (60.8)	<0.01	1955 (46.3)	476 (56.1)	<0.01
Male	6478 (54.1)	861 (41.1)		4210 (54.3)	488 (39.2)		2268 (53.7)	373 (43.9)	
Age at diagnosis									
Median (IQR)	77 (71–83)	75 (70–81)		75 (70–81)	74 (70–79)		80 (74–86)	77 (72–83)	
66–69	2017 (16.8)	424 (20.2)	<0.01	1569 (20.2)	299 (24.0)	<0.01	448 (10.6)	125 (14.7)	<0.01
70–74	2738 (22.8)	532 (25.4)		2031 (26.2)	342 (27.5)		707 (16.7)	190 (22.4)	
75–79	2625 (21.9)	499 (23.8)		1785 (23.0)	303 (24.3)		840 (19.9)	196 (23.1)	
80–84	2365 (19.7)	366 (17.5)		1398 (18.0)	194 (15.6)		967 (22.9)	172 (20.3)	
85+	2238 (18.7)	273 (13.0)		977 (12.6)	107 (8.6)		1261 (29.9)	166 (19.6)	
Marital status									
Married	4771 (39.8)	562 (26.8)	<0.01	3336 (43.0)	352 (28.3)	<0.01	1435 (34.0)	210 (24.7)	<0.01
Unmarried	3249 (27.1)	976 (46.6)		1906 (24.6)	542 (43.5)		1343 (31.8)	434 (51.1)	
Unknown	3963 (33.1)	556 (26.6)		2518 (32.4)	351 (28.2)		1445 (34.2)	205 (24.1)	
Region									
Northeast	4925 (41.1)	753 (36.0)	<0.01	3169 (40.8)	458 (36.8)	<0.01	1756 (41.6)	295 (34.7)	<0.01
Midwest	1211 (10.1)	269 (12.8)		808 (10.4)	147 (11.8)		403 (9.5)	122 (14.4)	
South	2276 (19.0)	775 (37.0)		1410 (18.2)	467 (37.5)		866 (20.5)	308 (36.3)	
West	3571 (29.8)	297 (14.2)		2373 (30.6)	173 (13.9)		1198 (28.4)	124 (14.6)	
Elixhauser score									
0	3632 (30.3)	513 (24.5)	<0.01	2635 (34.0)	327 (26.3)	<0.01	997 (23.6)	186 (21.9)	<0.01
1–2	4664 (38.9)	762 (36.4)		3112 (40.1)	488 (39.2)		1552 (36.8)	274 (32.3)	
3+	3687 (30.8)	819 (39.1)		2013 (25.9)	430 (34.5)		1674 (39.6)	389 (45.8)	
Frailty									
No	7786 (65.0)	1150 (54.9)	<0.01	5493 (70.8)	751 (60.3)	<0.01	2293 (54.3)	399 (47.0)	<0.01
Yes	4197 (35.0)	944 (45.1)		2267 (29.2)	494 (39.7)		1930 (45.7)	450 (53.0)	
Part D									
Never	3754 (31.3)	576 (27.5)	<0.01	1850 (23.8)	286 (23.0)	0.50	1904 (45.1)	290 (34.2)	<0.01
Ever	8229 (68.7)	1518 (72.5)		5910 (76.2)	959 (77.0)		2319 (54.9)	559 (65.8)	
State buy‐in									
No	11,002 (91.8)	1468 (70.1)	<0.01	7185 (92.6)	866 (69.6)	<0.01	3817 (90.4)	602 (70.9)	<0.01
Yes	981 (8.2)	626 (29.9)		575 (7.4)	379 (30.4)		406 (9.6)	247 (29.1)	
% below poverty in census tract									
0%– < 5%	3103 (25.9)	179 (8.5)	<0.01	2062 (26.6)	108 (8.7)	<0.01	1041 (24.7)	71 (8.4)	<0.01
5%– < 10%	3372 (28.1)	286 (13.7)		2215 (28.5)	183 (14.7)		1157 (27.4)	103 (12.1)	
10%– < 20%	3103 (25.9)	541 (25.8)		1990 (25.6)	334 (26.8)		1113 (26.4)	207 (24.4)	
20%–100%	1409 (11.8)	994 (47.5)		846 (10.9)	562 (45.1)		563 (13.3)	432 (50.9)	
Unknown	996 (8.3)	94 (4.5)		647 (8.3)	58 (4.7)		349 (8.3)	36 (4.2)	

*Note*: *p*‐values were from Pearson's χ^2^ test.

Abbreviations: IQR, interquartile range; NHAA, non‐Hispanic African American; NHW, non‐Hispanic White.

**FIGURE 2:**
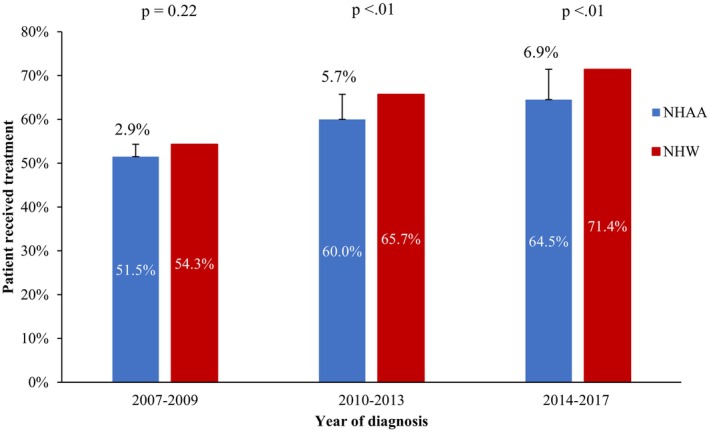
Treatment in the first year by race and year of diagnosis among 14,077 newly diagnosed multiple myeloma patients, 2007–2017.

During the first year following MM diagnosis, 59.5% of the NHAA patients and 64.8% of the NHW patients received treatment (*p* < 0.01). Among the NHAA group, patients who received treatment within 1 year increased from 51.5% in 2007–2009 to 64.5% in 2014–2017 (Figure 2, *p*_trend_ <0.01); among the NHW group, the percentages were 54.3% to 71.4%, respectively (*p*_trend_ <0.01). The discrepancy in the proportion of treated patients in the first year between the two racial groups did not reach statistical significance in 2007–2009 (p = 0.22) but did so in both 2010–2013 and 2014–2017 (both ps <0.01).

**FIGURE 3:**
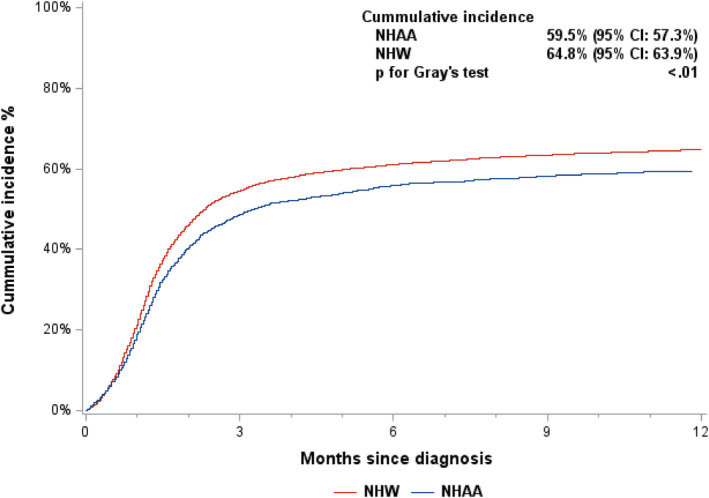
Cumulative incidence of receiving treatment among 14,077 newly diagnosed multiple myeloma patients, 2007–2017, by race.

As the cumulative probability of receiving treatment (Figure 3) indicated, treatments for NHAA patients were delayed, compared to NHW patients. After adjusting for baseline characteristics, treatment initiation was still significantly later among NHAA patients than in NHW patients (HR = 0.91, 95% CI: 0.85–0.97, *p* < 0.01). Treatment initiation was earlier for patients diagnosed in more recent periods (2014–2017: HR = 1.54, 95% CI: 1.46–1.63; 2010–2013: HR = 1.37, 95% CI: 1.30–1.44) than for those diagnosed in 2007–2010.

**FIGURE 4:**
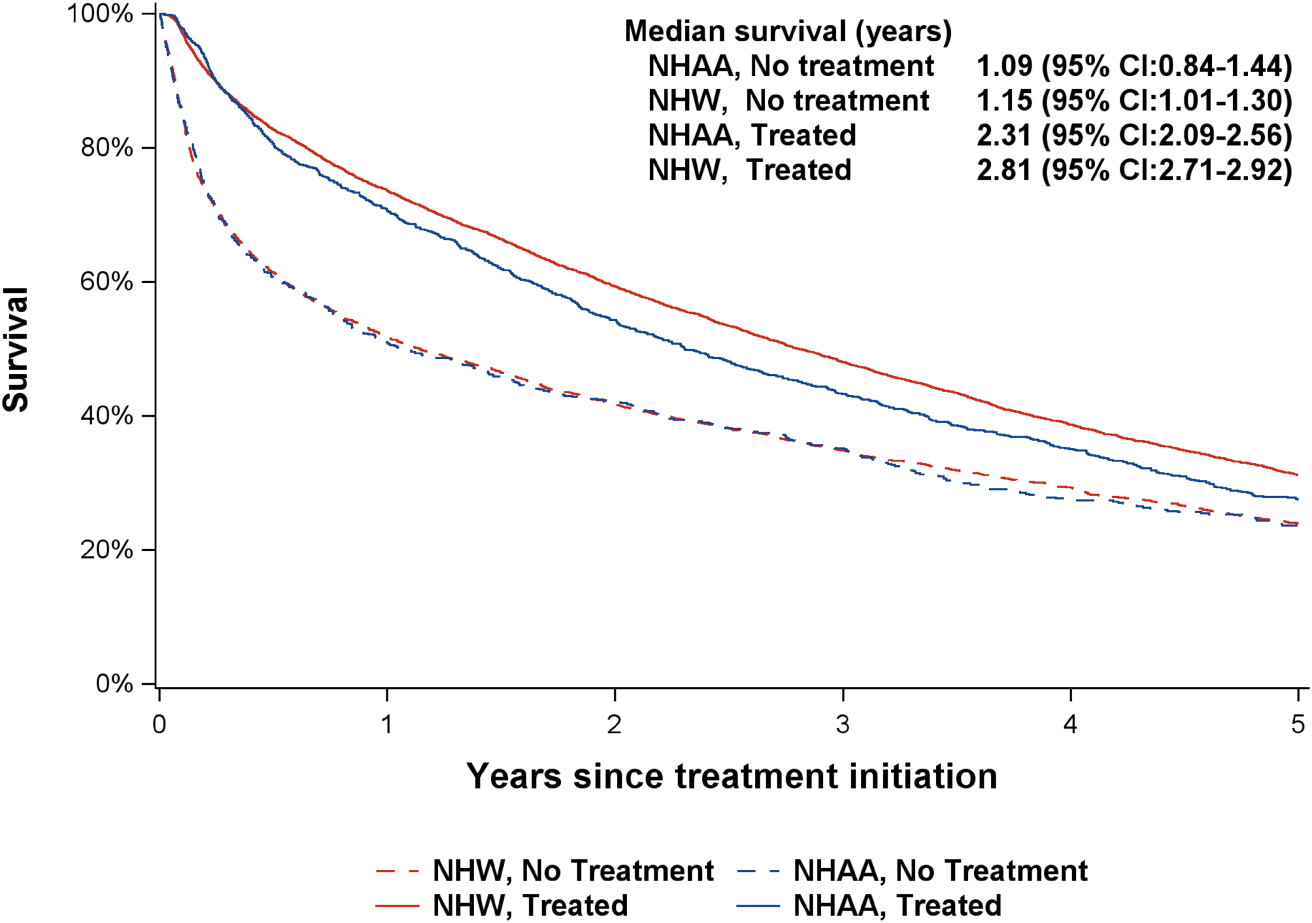
Kaplan–Meier curves of survival by treatment status within 1 year and race among 13,365 newly diagnosed multiple myeloma patients, 2007–2017*. *For patients who did not receive treatment, time zero was derived from the pseudo‐treatment initiation date.

In the univariate analysis, NHAA patients (1.97 years, 95% CI: 1.78–2.17) had shorter median survival (from the treatment/pseudo‐treatment date to death) than their NHW counterparts (2.37 years, 95% CI: 2.26–2.44, *p* for log‐rank <0.01). Patients who were treated in the first year (2.74 years, 95% CI: 2.65–2.85) had longer median survival than those who did not (1.13 years, 95% CI: 1.01–1.28, *p* for log‐rank <0.01). Among treated patients, NHW patients had longer median survival than their NHAA counterparts (*p* < 0.01, Figure 4). Yet among patients who did not receive treatment, no difference between the two racial groups was observed (*p* = 0.84). The median survival (from the treatment/pseudo‐treatment date to death) increased from 1.83 (95% CI: 1.68–1.95) years among patients diagnosed in 2007–2009 to 2.83 (95% CI: 2.67–3.00) years among those diagnosed in 2014–2017 (*p* < 0.01). In the multivariable model, compared to NHW patients, NHAA patients had lower 5‐year mortality (HR = 0.94, 95% CI: 0.88–1.00, *p* = 0.047, Table 2). Treatment decreased risk of death by 10% (HR = 0.90, 95% CI: 0.86–0.94). In contrast to individuals diagnosed in 2007–2009, those diagnosed in more recent years exhibited a reduced risk of death (2010–2013: HR = 0.86, 95% CI 0.82–0.91; 2014–2017: HR = 0.78, 95% CI 0.74–0.82). No statistically significant interaction between race and year of diagnosis was observed (*p* = 0.92).

**TABLE 2:** Adjusted hazard ratios of mortality among patients with newly diagnosed multiple myeloma, overall and by treatment received status.

	Overall^a^		Treated^b^		No treatment^b^	
	HR (95% CI)	*p*	HR (95% CI)	*p*	HR (95% CI)	*p*
Non‐Hispanic African American	0.94 (0.88–1.00)	0.05	0.98 (0.90–1.06)	0.63	0.84 (0.77–0.93)	<0.01
Treatment	0.90 (0.86–0.94)	<0.01				
Year of diagnosis						
2007–2009	1.00		1.00		1.00	
2010–2013	0.86 (0.82–0.91)	<0.01	0.85 (0.80–0.91)	<0.01	0.93 (0.86–1.00)	0.05
2014–2017	0.78 (0.74–0.82)	<0.01	0.75 (0.70–0.80)	<0.01	0.92 (0.85–0.99)	0.04
Part D	0.63 (0.61–0.66)	<0.01	0.65 (0.61–0.69)	<0.01	0.64 (0.60–0.69)	<0.01
Male	1.14 (1.09–1.20)	<0.01	1.16 (1.10–1.23)	<0.01	1.09 (1.02–1.17)	0.01
Age at diagnosis						
66–69	1.00		1.00		1.00	
70–74	1.22 (1.14–1.32)	<0.01	1.29 (1.19–1.41)	<0.01	1.07 (0.93–1.22)	0.34
75–79	1.46 (1.36–1.57)	<0.01	1.56 (1.43–1.70)	<0.01	1.25 (1.10–1.42)	<0.01
80–84	1.91 (1.77–2.05)	<0.01	1.94 (1.78–2.12)	<0.01	1.69 (1.49–1.92)	<0.01
85+	2.58 (2.39–2.78)	<0.01	2.55 (2.32–2.81)	<0.01	2.21 (1.96–2.50)	<0.01
Marital status						
Married	1.00		1.00		1.00	
Unmarried	1.22 (1.15–1.28)	<0.01	1.15 (1.08–1.23)	<0.01	1.29 (1.19–1.40)	<0.01
Unknown	0.92 (0.87–0.98)	0.01	0.89 (0.82–0.96)	0.00	1.03 (0.93–1.13)	0.62
Region						
Northeast	1.00		1.00		1.00	
Midwest	1.07 (0.98–1.15)	0.13	1.03 (0.93–1.14)	0.60	1.21 (1.07–1.36)	<0.01
South	0.98 (0.91–1.05)	0.57	0.96 (0.88–1.05)	0.39	1.05 (0.94–1.17)	0.42
West	0.94 (0.88–1.00)	0.05	0.94 (0.87–1.01)	0.09	0.96 (0.88–1.06)	0.46
Elixhauser score						
0	1.00		1.00		1.00	
1–2	1.05 (1.00–1.11)	0.07	1.09 (1.02–1.16)	0.01	1.00 (0.91–1.09)	0.96
3+	1.42 (1.33–1.51)	<0.01	1.47 (1.36–1.59)	<0.01	1.39 (1.26–1.54)	<0.01
Frailty	1.28 (1.21–1.35)	<0.01	1.26 (1.18–1.34)	<0.01	1.29 (1.19–1.40)	<0.01
% below poverty in census tract						
0%–<5%	1.00		1.00		1.00	
5%–<10%	1.09 (1.02–1.16)	0.01	1.08 (1.00–1.16)	0.04	1.12 (1.02–1.24)	0.02
10%–<20%	1.14 (1.07–1.22)	<0.01	1.12 (1.03–1.21)	0.01	1.20 (1.09–1.32)	<0.01
20%–100%	1.23 (1.15–1.33)	<0.01	1.21 (1.10–1.33)	<0.01	1.26 (1.13–1.41)	<0.01
Unknown	1.16 (1.06–1.28)	<0.01	1.10 (0.97–1.24)	0.13	1.23 (1.06–1.43)	0.01
State buy‐in	1.38 (1.29–1.48)	<0.01	1.30 (1.19–1.42)	<0.01	1.53 (1.38–1.69)	<0.01

*Note*: All variables in the table were mutually adjusted in the Cox Proportional hazard regression models.

Abbreviations: CI, confidence interval; HR, hazard ratio.

^a^ For overall, we followed patients from treatment initiation date or pseudo‐initiation date. A total of 712 patients who did not received treatment during the first year after diagnosis and died before the pseudo‐treatment initiation date were excluded for the analysis.

^b^ For analyses stratified by treatment status, we followed patients from date of multiple myeloma diagnosis.

In the separate analysis stratified by treatment status, after accounting for patient's sociodemographic characteristics, we found that NHAA and NHW patients who received treatment had similar survival (HR = 0.98, 95% CI: 0.90–1.06, *p* = 0.63, Table 2), whereas NHAA patients without any treatment in the first year had a reduced risk of death than their NHW counterparts (HR = 0.84, 95% CI: 0.77–0.98, *p* < 0.01). Furthermore, the year of MM diagnosis emerged as a predictor of survival for treated patients but did not exhibit a significant association with survival among non‐treated patients.

## 4 DISCUSSION

In this study, we found that among older patients with symptomatic MM, despite the availability of therapeutical choices and updated guidelines recommending the use of these agents, there was a persistent racial difference in treatment received and timing of treatment initiation. We observed no racial difference in survival among treated patients, which was consistent with findings from patients who were enrolled in clinical trials.^17^ NHAA patients had better survival than NHW patients among those who did not receive treatment during the first year. Collectively, these findings indicated that biological differences in MM may exist by race. For example, analyzing VA data, researchers discovered NHAA patients had a lower prevalence of the Del17p mutation, a genetic abnormality linked to poor prognosis, compared to NHW patients.^18^ Some studies even found that when taking treatments into consideration, NHAA patients had better survival than NHW patients.^19^, ^20^, ^21^ Fillmore et al. reported that in the veteran population, who had relatively equal access to care compared to the general population, NHAA patients aged <65 years had better survival than their NHW counterparts.^21^ In addition, a SEER‐Medicare study found that when treated equally, NHW patients had 13% excess risk of 5‐year death than that of matched NHAA patients.^19^ Thus, the lack of racial difference in MM survival that we observed among MM patients who received treatment may have resulted from the racial inequity in timing of treatment initiation and treatment adherence.

Consistent with previous studies, NHAA patients were consistently less inclined to undergo treatment and tended to experience a delay in treatment initiation.^7^, ^8^, ^9^, ^22^ While the proportion of patients receiving treatment increased over time in both racial groups, a growing gap between NHAA and NHW patients (2.9% in 2007–2009 vs. 6.9% in 2014–2017) is concerning. Prior literature has demonstrated that among SEER‐Medicare MM patients diagnosed in 2007–2013 and followed to the end of 2014, the utilization of novel agents increased in both NHAA and NHW patients, yet the increasing trend was more notable in the NHW cohort compared with NHAA cohort.^9^ The gap may be related to the affordability of costly medications for MM treatment, such as lenalidomide.^23^, ^24^, ^25^ These medications are administered orally and covered by Medicare Part D program. The financial burden associated with oral agents through out‐of‐pocket liability is substantial and would make it difficult for some NHAA patients to continue maintenance therapy. Moreover, the financial burden would increase over time given that the average net price of one lenalidomide pill increased from $215 in 2005 to $719 in 2019,^23^ and the estimated median annual out‐of‐pocket cost of lenalidomide alone for patients with Part D coverage increased by $2923 from $11,538 in 2016^26^ to $14,461 in 2019.^24^

Aligning with prior studies, our study also found that NHAA patients tended to initiate treatment later than NHW patients. Among patients diagnosed in 2007–2013, it took a median of 5.2 months for NHAA patients and 2.7 months for NHW patients to initiate novel therapy.^9^ While Ailawadhi et al. and our study had the same conclusion, our study found that the median times to treatment initiation in both racial groups were shorter than those in Ailawadhi et al, which potentially indicates practice change in MM treatment. The association between the year of diagnosis and timing of treatment initiation also demonstrated the improvement in real‐world practice. As a claim‐based study, however, we were unable to identify reasons for the initiation delay. It is important to provide timely guideline‐recommended treatment regardless of race. Understanding the causes of differences in treatment initiation and the associations between timing of treatment initiation and survival outcomes could help inform interventions to reduce racial inequity in MM care.

We observed a more prominent association between diagnosis year and MM survival in patients underwent treatment than in those patients without treatment. These findings are important for understanding disease trajectory of MM in the United States. As MM treatments evolved over time, and the novel agents were more effective than the old ones, patients who received treatment could benefit from these new medications. Second, it appeared that the year of diagnosis did not have considerable impact on survival outcomes for patients who did not receive treatment, indicating that, at the population level, the natural history of MM progression did not change substantially over time once MM became symptomatic.

In our study, we found NHAA patients were less likely to die than NHW patients in the overall and those who did not receive treatment within the first year, but no racial differences in survival between those who received treatment. The lack of survival advantages among treated NHAA patients might be the result of differences in treatment initiation between the two racial groups. Our analyses demonstrated that NHAA patients were less likely to initiate treatment, compared to NHW patients. Future studies examining the association between timing of treatment initiation and survival are needed.

There are several limitations in this study. First, the SEER‐Medicare database lacked comprehensive clinical, laboratory, and pathologic details which prevented us from evaluating disease stage, severity, or cytogenetic risk factors which may impact MM treatment choices and survival. Similarly, we are unable to determine the reasons for not receiving treatment or delaying treatment initiation. Second, the database did not include data on individual‐level socioeconomic status (except for state buy‐in as a proxy marker), even though we had access to poverty information at the census tract level. Third, our study population was derived from individuals aged ≥66 years with continuous Medicare fee‐for‐service coverage; thus, the results from our analyses may not be applicable to individuals covered by other insurances or younger patients.

In conclusion, despite the availability of many therapeutical options, there was an increasing trend of racial disparity in treatment utilization. As novel agents are effective but expensive, such inequity may escalate if financial burden is the primary barrier for NHAA patients with MM to access care. Although the Affordable Care Act prohibited lifetime monetary caps on insurance coverage and restricted the use of annual caps in 2010, inequities in MM care persist among the Medicare population. Whether the recent changes in policies/regulations, such as oncology care model and the Inflation Reduction Act, could reduce racial disparity is worth investigating. Nevertheless, in order to provide timely optimal and equal treatment access regardless of race, efforts are needed to eliminate the barriers of receiving treatment, especially for NHAA patients.

## AUTHOR CONTRIBUTIONS

**Rong Wang:** Formal analysis (equal); methodology (equal); writing – original draft (equal); writing – review and editing (equal). **Natalia Neparidze:** Methodology (equal); writing – review and editing (equal). **Xiaomei Ma:** Conceptualization (equal); methodology (equal); writing – review and editing (equal). **Graham A. Colditz:** Funding acquisition (equal); methodology (equal); writing – review and editing (equal). **Su‐Hsin Chang:** Conceptualization (equal); funding acquisition (equal); methodology (equal); project administration (equal); writing – review and editing (equal). **Shi‐Yi Wang:** Conceptualization (equal); funding acquisition (equal); methodology (equal); project administration (equal); supervision (equal); writing – review and editing (equal).

## CONFLICT OF INTEREST STATEMENT

Dr Neparidze reported receiving grants from Janssen and GlaxoSmithKline outside the submitted work. Dr Ma reported receiving consultation fees from Bristol Myers Squibb outside the submitted work. No other disclosures were reported.

## ETHICS STATEMENT

The Yale Human Investigation Committee determined that this study did not directly involve human subjects.

## Data Availability

The datasets used to conduct this study are available upon approval of a research protocol from the National Cancer Institute. Instructions for obtaining these data are available at https://healthcaredelivery.cancer.gov/seermedicare/obtain/.
